# Developments in smoking behaviour during the transition from adolescence to young adulthood. Results of the KiGGS cohort

**DOI:** 10.17886/RKI-GBE-2018-029

**Published:** 2018-03-15

**Authors:** Elvira Mauz, Benjamin Kuntz, Johannes Zeiher, Felicitas Vogelgesang, Anne Starker, Thomas Lampert, Cornelia Lange

**Affiliations:** Robert Koch Institute, Berlin, Department of Epidemiology and Health Monitoring

**Keywords:** SMOKING, TRANSITION, YOUNG ADULTS, HEALTH MONITORING, KIGGS COHORT

## Background

The consumption of tobacco products is regarded as the largest preventable risk factor for a large number of serious diseases. It causes around 121,000 deaths per year in Germany alone [1]. Over the last two decades, various tobacco control measures have been taken and extended over time such as increasing tobacco taxes, advertising bans and implementing age restrictions as well as smoking bans to protect non-smokers from second hand smoke exposure. Overall, tobacco consumption has decreased during this period [2] and the proportion of smokers among children and adolescents declined significantly. However, as a large proportion of the population continues to smoke, reducing tobacco consumption remains one of the key objectives of public health [3, 4]. Smoking in childhood and adolescence may still be experimental in character, but it often proves to be the beginning of a consumption pattern that is highly stable over the life course [5, 6]. In this context, this article uses longitudinal data from the KiGGS cohort to examine trajectories of smoking behaviour during the transition from adolescence to young adulthood.

## Indicator and methodology

The analyses presented here are based on self-reports on current smoking (any smoking, even occasional) and on the age of smoking initiation. The data is taken from the KiGGS cohort, a study that follows the participants of the KiGGS baseline study (2003-2006) [7] into adulthood. The sample includes 2,159 young adults (1,159 women; 1,000 men) aged 19 and 24 years old who have participated again in the first follow-up telephone survey of KiGGS Wave 1 (2009-2012). In total, 57.8% of the original 3,736 14 to 17 year-olds in the KiGGS baseline study with valid information on current smoking at both measurement times were included. Transition probabilities were calculated, that means the percentage probability of the transition from smoking to non-smoking or vice versa from the KiGGS baseline study to KiGGS Wave 1. Socioeconomic status (SES) was determined using the data provided by parents on education, occupation and income at the time of the KiGGS baseline study [8]. Possible bias caused by selective re-participation was partially taken into account through multivariate weighting [7].

**The KiGGS study**

The German Health Interview and Examination Survey for Children and Adolescents

**Data owner:** Robert Koch Institute

**Aim:** Providing reliable information on health status, health-related behaviour, living conditions, protective and risk factors, and health care among children, adolescents and young adults living in Germany, with the possibility of trend and longitudinal analyses

**Study design**: Combined cross-sectional and cohort study

**KiGGS survey waves**

► KiGGS baseline study (2003-2006), examination and interview survey
► KiGGS Wave 1 (2009-2012), interview survey
► KiGGS Wave 2 (2014-2017), examination and interview survey

**KiGGS cross-sectional study**

**Population:** Children and adolescents with permanent residence in Germany

**Age range:** 0-17 years

**KiGGS cohort study**

**Sampling:** Re-invitation of everyone who took part in the KiGGS baseline study (n=17,641) and who was willing to participate in a follow-up

**Age range KiGGS Wave 1:** 6-24 years (n=11,992)

**Age range KiGGS Wave 2:** 10-31 years (n=10,853)

More information is available at www.kiggs-studie.de/english

## Results

The proportion of people in the sample who smoked increased from 32% to 43% between the periods during which the KiGGS baseline study and KiGGS Wave 1 were undertaken. 85% of adolescents who smoked and 78% of non-smoking adolescents did not change their smoking behaviour in young adulthood. Whereas 15% of young people who smoked quit smoking between the two survey periods, 22% of non-smokers starting smoking during this time. The analyses of data covering the age when a respondent began to smoke show that almost nine out of ten participants who ever smoked started smoking before the age of 18.

**Figure 1:**
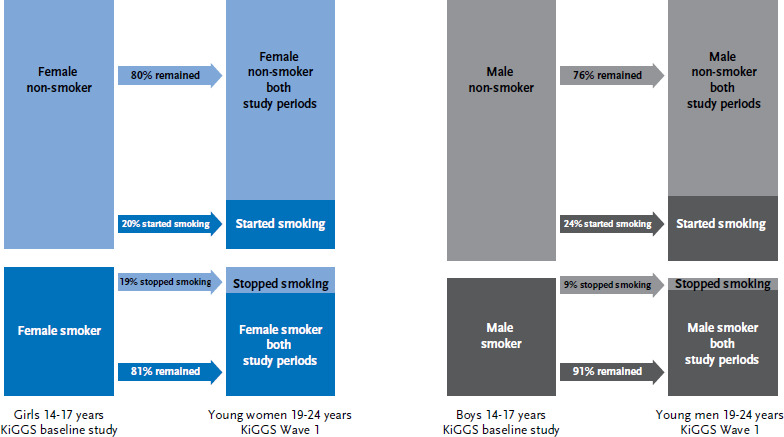
Developments in smoking behaviour during the transition from adolescence to young adulthood (n=1,159 female, n=1,000 male) Source: KiGGS baseline study (2003-2006), KiGGS Wave 1 (2009-2012)

Although no differences were found between the proportions of girls and boys who smoked during adolescence, the proportion of smoking in young adulthood increases more considerable in young men than in women. The main reason for this difference is that women who smoked as adolescents quit smoking significantly more often than men (19% vs. 9%) during the transition into adulthood. At the same time, formerly non-smoking male adolescents have started smoking more often; however, this difference is not statistically significant (Figure 1).

Finally, there is a strong correlation between SES and smoking behaviour. For both survey periods, a larger proportion of people in the low status group smoked than in the high status group (KiGGS baseline study: 37% vs. 23%; KiGGS Wave 1: 49% vs. 33%). Non-smoking adolescents with a low SES started smoking more often as young adults, and young smokers with a low SES quit smoking slightly less frequently than those with a high SES. However, these social differences of individual changes are not statistically significant.

## Discussion

The results demonstrate that smoking behaviour remains relatively stable during the transition from adolescence to young adulthood; this finding is in line with the existing literature [6]. A large proportion of non-smoking adolescents do not start smoking during the transition into adulthood; nevertheless, only a minor proportion of the smoking adolescents quit smoking during young adulthood. These figures illustrate the importance of preventing children and adolescents from starting to smoke. In addition, the results indicate that the social differences in smoking behaviour that are already evident among adolescents become stabilised in later life, and therefore contribute to long-term health inequalities. However, it has to be acknowledged that smoking participants with a low SES more often did not take part in KiGGS Wave 1. This may have led to an underestimation of prevalence. It was not possible to completely offset this dropout using weighting.

Trend analyses of the cross-sectional data from KiGGS show that smoking prevalence rates among adolescents in Germany have steadily decreased over the past ten years [3]. However, the results set out here demonstrate that on an individual level the smoking behaviour of the formerly adolescent participants of the KiGGS cohort remains relatively stable during the transition into adulthood; most people who smoked during young adulthood started this behaviour during adolescence. These results illustrate the added value of the longitudinal data gained by continuation of the KiGGS cohort. Future analyses should aim to identify influencing factors that prevent or encourage smoking and that also promote or make it more difficult to quit. The results of such analyses could then be used to develop target group-specific interventions in tobacco prevention and smoking cessation or rather to evaluate the measures that are currently in place.
